# Measurement of Nanowire Optical Modes Using Cross-Polarization Microscopy

**DOI:** 10.1038/s41598-017-18193-1

**Published:** 2017-12-19

**Authors:** Joona-Pekko Kakko, Antti Matikainen, Nicklas Anttu, Sami Kujala, Henrik Mäntynen, Vladislav Khayrudinov, Anton Autere, Zhipei Sun, Harri Lipsanen

**Affiliations:** Department of Electronics and Nanoengineering, Aalto University, P.O.Box 13500, FI-00076 Aalto, Finland

## Abstract

A method to detect optical modes from vertical InGaAs nanowires (NWs) using cross-polarization microscopy is presented. Light scattered from the optical modes in the NWs is detected by filtering out the polarized direct reflection with a crossed polarizer. A spectral peak and a valley were seen to red-shift with increasing NW diameter in the measured spectra. The peak was assigned to scattering from the TE_01_ optical mode and the valley was an indication of the HE_11_ mode, based on finite-element and scattering matrix method simulations. The cross-polarization method can be used to experimentally determine the spectral positions of the TE_01_ and HE_11_ optical modes. The modes are significantly more visible in comparison to conventional reflectance measurements. The method can be beneficial in the characterization of NW solar cells, light-emitting diodes and lasers where precise mode control is required.

## Introduction

Semiconductor nanowires (NWs) have been proposed to be used as efficient solar cells^1–4^, light-emitting diodes (LEDs)^5,6^ and lasers^7,8^ due to their large active area compared to volume. Also, NWs have the ability to enhance coupling of light and allow directional and polarized scattering at resonant wavelengths^9–12^. The optical design of NW solar cells, LEDs and lasers is typically based on simulations to optimize the NW dimensions in order to match the resonant wavelength to the solar spectrum or to the desired emission wavelength range. To verify the match experimentally and to study the performance, electrical and/or spectral photocurrent measurements are usually needed^2,10,13^. To be able to perform these measurements, a fully processed device is needed. Such a path can be cumbersome and costly as many delicate processing steps are required.

Reflectance spectroscopy has emerged as an important tool for the optical characterization of NWs. Unlike imaging, reflectance is not limited by diffraction. Thus, sub-wavelength information, such as diameter and length of the NWs, can be acquired from the spectra by associating spectral features to simulations or to spectra of known samples. However, complicated interpretation of the reflectance spectra is required to be able to do this^13–16^.

In this paper, a method to detect the optical modes from vertical InGaAs NW arrays with a reflectance setup in cross-polarization configuration is presented and compared to conventional reflectance spectroscopy. With this cross-polarization method, the directly reflected light from the NWs and substrate is filtered out using a polarizer, allowing the detection of scattered light only from the NWs. By using finite-element method and scattering matrix method simulations, the scattered light can be attributed to light interacting mainly with the TE_01_ optical mode.

Earlier, scattered light and the subsequent characterization of optical modes from dispersed horizontal NWs have been studied with dark-field microscopy^17–19^. In comparison, here the optical modes are characterized directly from NW arrays vertically standing on the substrate without additional processing. The method can be used to experimentally identify the TE_01_ optical mode in the NWs. The information could then be used to estimate the positions of other optical modes, and it is beneficial when NWs are to be used in solar cells, LEDs or in other optoelectronical applications where the optimization of the optical modes is essential.

## Methods

### Sample fabrication

The InGaAs NW arrays were fabricated on a GaAs (111)B substrate using atmospheric pressure selective-area metalorganic vapour phase epitaxy (MOVPE). Initially, a 40 nm layer of SiO_*x*_ was deposited on the clean substrate using plasma-enhanced chemical vapour deposition (PECVD) with an Oxford Systems Plasmalab 80 Plus system. Next, electron beam lithography (EBL) was used to pattern several 100 *μ*m by 100 *μ*m arrays of holes. The arrays of holes were arranged into triangular lattice with array periodicity varying from 250 nm to 6 *μ*m, with a minimal step of 25 nm. Diameters of the holes were varied from 40 nm to 70 nm with a 5 nm step. Polymethyl methacrylate (PMMA, 950 kg/mol, 2% in anisole, from Microchem) was used as the EBL resist and a Vistec EPBG5000pES system was used for the EBL itself. The EBL pattern was transferred to the SiO_*x*_ layer with reactive-ion etching using identical system as for the PECVD.

The patterned sample was transferred into the MOVPE reactor (1″ horizontal flow reactor, Thomas Swan) after cleaning and degreasing in acetone, isopropanol and de-ionized water. Prior to NW growth, the sample was annealed at 750 °C in the reactor under tertiarybutyl arsine (TBAs) flow for 5 min. The selective-area growth of the NWs was initiated by lowering the temperature to 740 °C and turning on the flow of trimethylgallium (TMGa) and trimethylindium (TMIn) in addition to the TBAs flow for 10 min. After the growth step, the sample was cooled down to room temperature under TBAs flow. H_2_ was used as the carrier gas and the total gas flow was kept constant at 5 slm. The flow rates of TBAs, TMGa and TMin were 54.0 *μ*mol/min, 0.81 *μ*mol/min, and 0.31 *μ*mol/min, respectively.

### Optical spectroscopy

**Figure 1:**
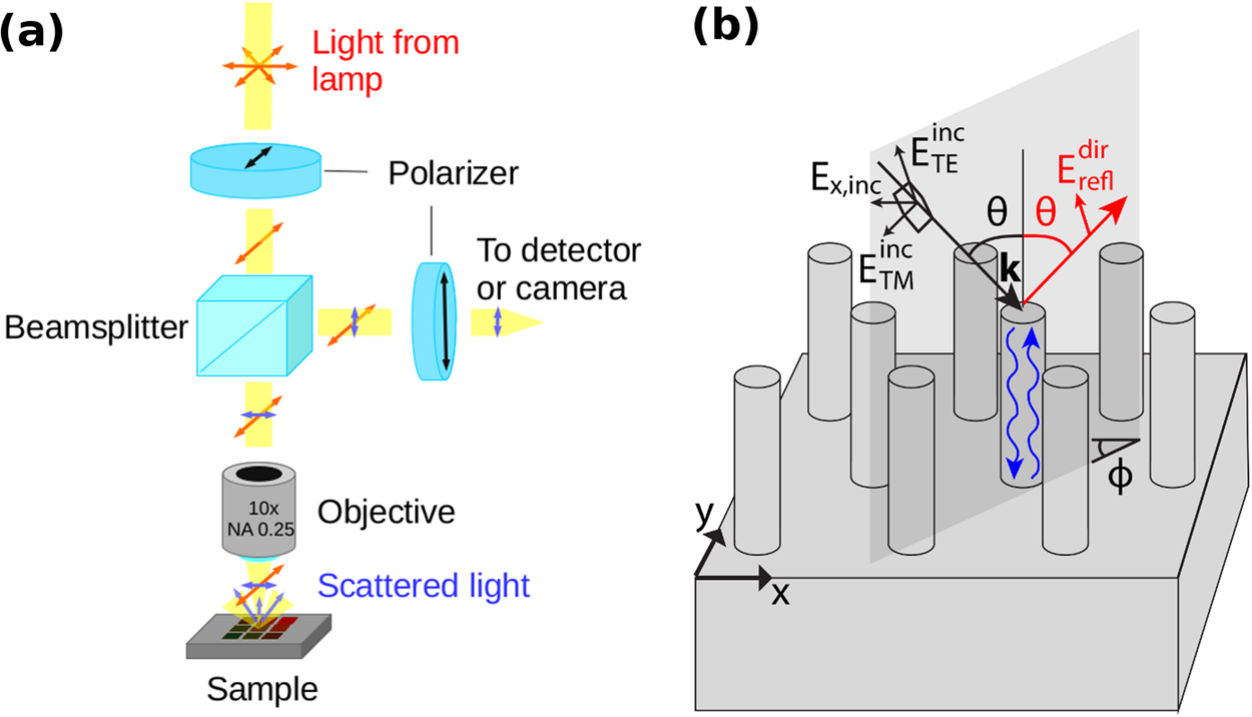
(**a**) Schematic presentation of the optical microscopy setup. Polarized light is focused on the sample using a 10x microscope objective. A second polarizer is used to filter out the direct reflection in order to detect only the scattered light originating from the NWs. (**b**) Schematic of the NW array with definition of polar and azimuth angles *θ* and *ϕ*. The shaded plane indicates the incidence plane which, by definition, contains the k-vector of the incident light and the surface normal of the substrate, which is parallel to the NW axis. Here, TE (TM) polarized incident light has no electric (magnetic) field component in the incidence plane. Also, the x-component of the incident light in the cross-polarization measurements, a possible change of the polarization direction in the directly reflected light (red), and coupling to optical modes (blue) in the NWs have been indicated.

A conventional microscope setup was used to perform the optical measurements and imaging. The setup is depicted in Fig. 1(a). For the cross-polarization measurements, one Glan-Taylor polarizer was used to polarize the incident light and a second one to cross-polarize the reflected light. A xenon lamp was used for the illumination and a 10x microscope objective (NA 0.25) was used to focus the light on the sample. This resulted in an approximately 60 *μ*m diameter spot size. An Ocean Optics HR4000 spectrometer was used to record the spectra. The unpatterned sample area was used as the reference (SiO_*x*_/GaAs). Background spectrum was substracted from the spectra before any further processing. The cross-polarization spectrum was obtained by dividing the raw measured spectrum with the raw reference spectrum in order to see relative change in the NWs. Reflectance spectra were measured in a similar way, but with a less intense halogen lamp for illumination and without polarizers.

Note that in theory, when using cross-polarization configuration, the signal from a blank reference should be nil. However, due to nonidealities, a small amount of light is always transmitted. This was used to establish a detection limit for the experiments. When actual scattered light with crossed polarization is detected, its intensity is higher than the reference baseline.

### Scattering matrix method simulations

The simulated cross-polarization and reflectance spectra were acquired by solving the Maxwell equations with scattering matrix method (SMM)^20^ for 3000 nm long circular InGaAs NWs placed in a triangular lattice on a GaAs substrate with 40 nm thick SiO_2_ on the substrate, between the NWs. The diameters of the NWs used for the modeling were taken from a line fitted to measured diameters as a function of array period (Supporting Information Figure S1). Tabulated values of the refractive indices for GaAs^21^ and SiO_2_^22^ were used. The wavelength dependent complex refractive index for InGaAs was used from ref.^23^.

The modeling is performed similarly as in ref.^24^ for a finite numerical aperture for illumination and collection of reflection, but here, an explicit analysis of the cross-polarization is included. The model definitions for the polar angle *θ*, azimuth angle *ϕ* and polarization are illustrated in Fig. 1(b). For the modeling of cross-polarization, the incident light is *x*-polarized and contributions from *x*- and *y*-polarized light are studied separately in reflection (with y-polarized reflected light giving the cross-polarization). The NA of 0.25 corresponds to a polar angle of *θ* = arcsin(0.25) ≈ 14°. Thus, contribution of reflected light propagating at an angle up to this angle is included in the shown spectra. The modeling was performed for incidence polar angle *θ* ≤ arcsin(0.25) and results were averaged over *ϕ*.

### Finite-element method simulations

The mode analysis was performed using 2D finite element method (FEM) simulation with a commercial COMSOL V5.2 software package. The mode fields were calculated for infinite hexagonal InGaAs nanowires with diameters from 80 to 200 nm at 5 nm intervals and the wavelength from 400 to 850 nm at 12.5 nm intervals. The refractive index data was same as above. The simulations were carried out on single air-clad NWs surrounded by a perfectly matched layer (12 *μ*m × 12 *μ*m grid size). Note that the fundamental HE_11_ mode stays guided for all wavelengths^25,26^. In contrast, the other modes show for a given diameter a cut-off wavelength above which the mode becomes a leaky mode^25,26^.

## Results and Discussion

### Nanowire characterization

**Figure 2:**
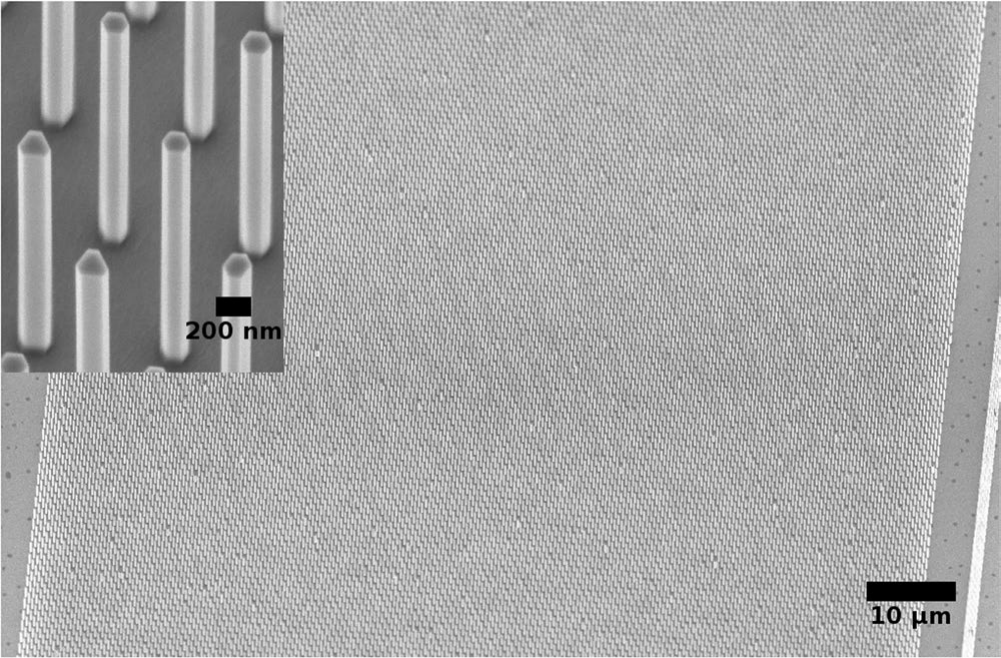
25° tilted general view and magnified (inset) SEM images of InGaAs NW arrays with 800 nm array period. The NWs are vertical and untapered and the arrays have low defect densities.

Figure 2 shows scanning electron microscope (SEM) images of the resulting InGaAs NW arrays with a pitch of 800 nm. The NW arrays have low defect densities and the NWs are vertical and untapered, as seen in the SEM images. The composition of the NWs was confirmed to be In_0.38_ Ga_0.62_ As with energy-dispersive X-ray (EDX) analysis from dispersed NWs on SiO_2_/Si. The heights of the NWs are between 2.5 *μ*m and 3.5 *μ*m, estimated from tilted SEM images. The diameters of the NW arrays were measured from top-view SEM images. The long diagonal of the hexagonal cross-section was taken as the diameter in this work. The average diameters of the arrays varied from approximately 75 nm to 200 nm, increasing with the array period (see Supporting Information Figure S1).

### Cross-polarization imaging

**Figure 3:**
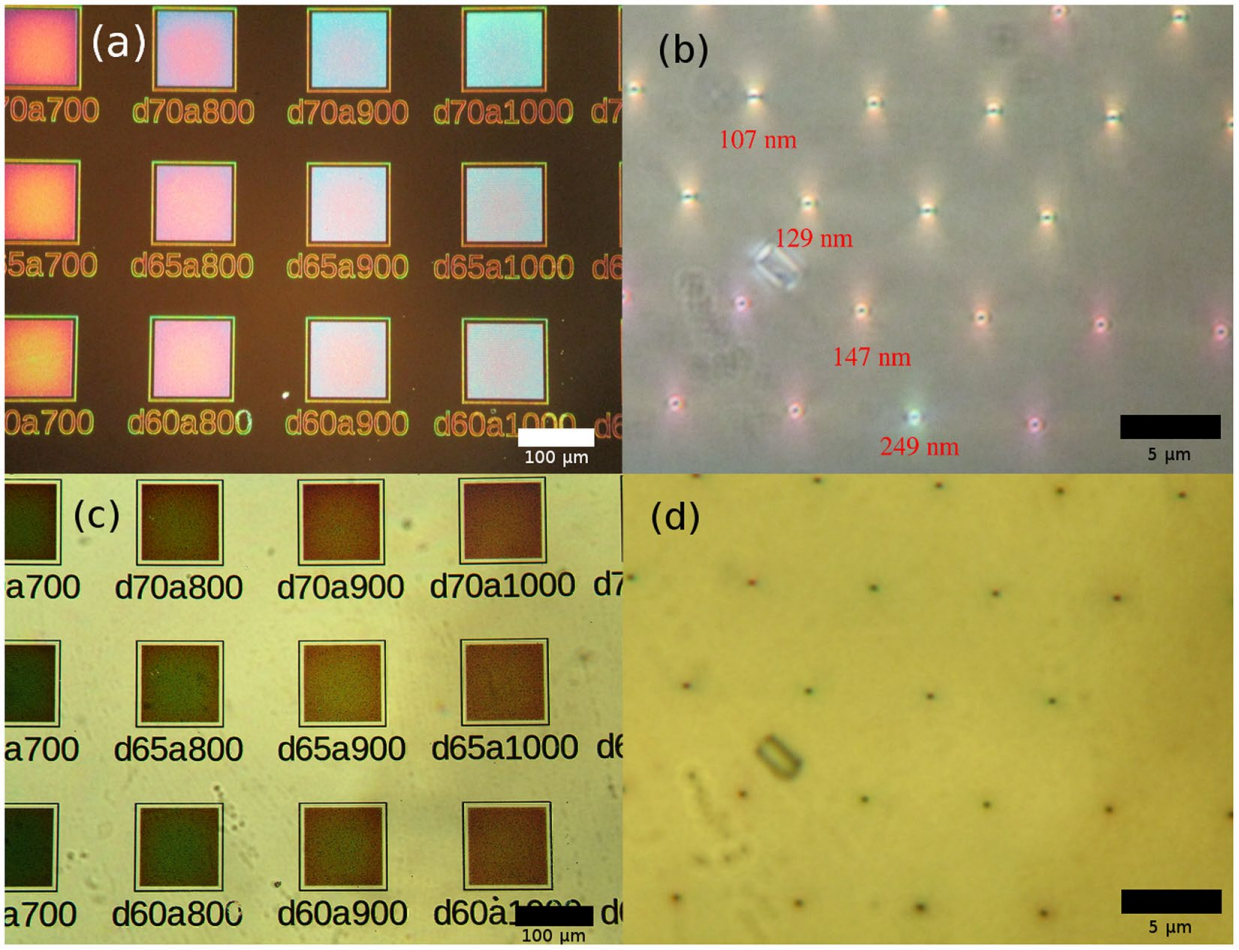
Low (**a**) and high (**b**) magnification cross-polarization microscope images showing vivid colors from NW arrays with different dimensions. Corresponding low (**c**) and high (**d**) magnification bright-field microscope images of the same areas. Cross-polarization imaging reveals optical modes that scatter light, hence strong color contrast and pattern change is seen even from single NWs. The approximate diameters of selected NWs are labeled in red below them in (**b**). The text in the low magnification images is NW array labeling.

Figure 3 shows low and high magnification optical microscope images of the vertical InGaAs NW arrays in cross-polarization configuration (a,b) and in bright-field configuration (c,d). Color changes with varying array period and NW diameter are observed in both configurations from different NW arrays. However, the colors are distinctively different with the two methods. Different colors even for single NWs with large spacing can be easily distinguished in cross-polarization (b) with varying NW diameter, unlike in bright-field (d). Surprisingly, in addition to the change of the color from single NWs in cross-polarization mode, an accompanying halo-like pattern around the individual NWs is observed in high magnification. For small diameters, a two-lobe pattern shows up, which changes into a donut shape as the diameter increases. This change could be an indication of contribution from different optical modes in NWs of varying diameter.

The perceived color of the NWs is governed by the coupling of incident light into the optical modes in them. In direct reflection, the color observed is due to combined scattering and reflection from both the NWs and substrate^18,24,27^. In the cross-polarization mode, the color observed is the light scattered by the NWs only. Furthermore, the color of the NWs in cross-polarization mode did not change with different NA values (see Supporting Information Figure S2).

### Cross-polarization spectroscopy and comparison to reflectance

**Figure 4:**
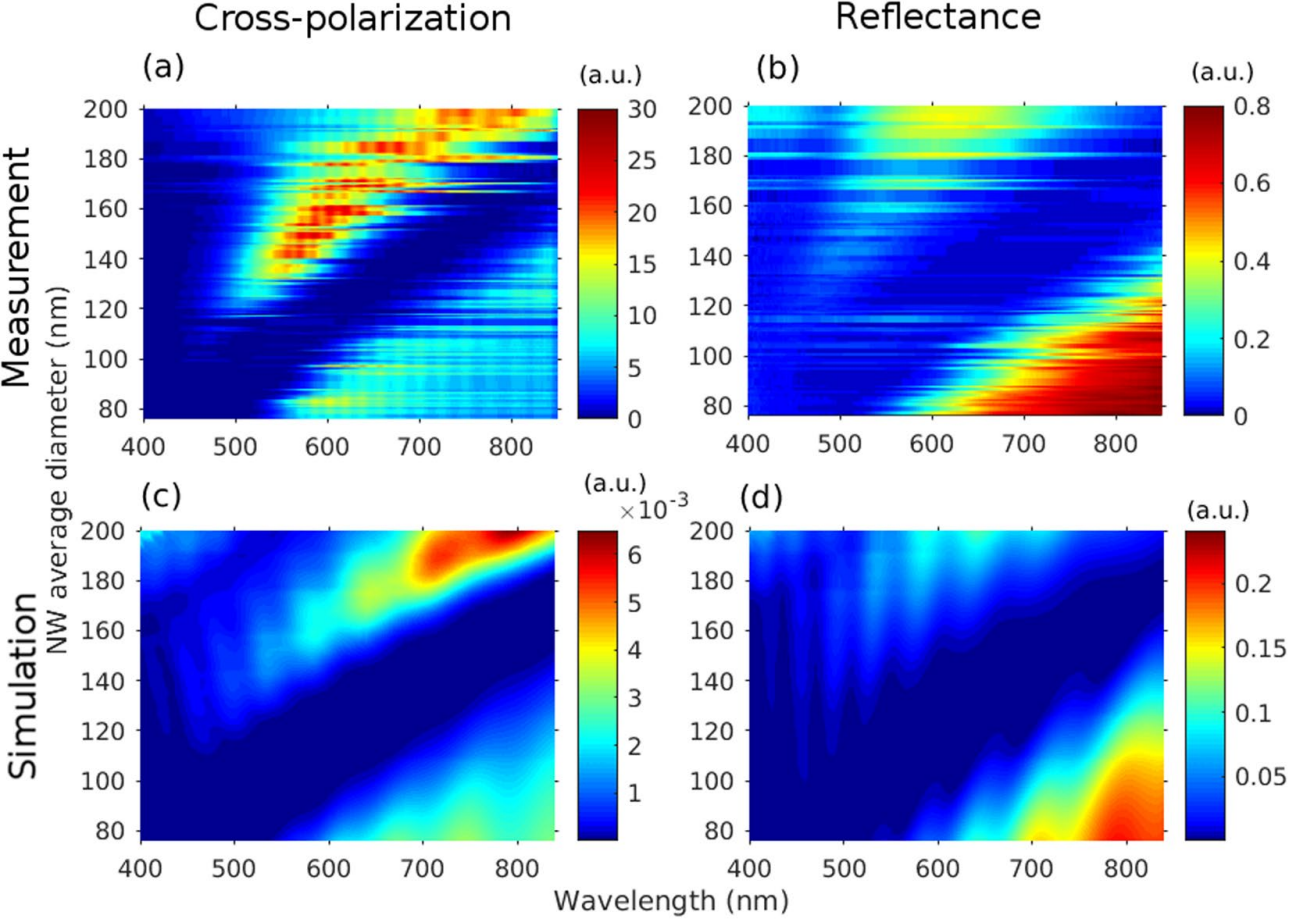
Intensity color maps of the measured cross-polarization and reflectance spectra (**a**,**b**) with varying NW average diameter and the corresponding simulated spectra (**c**,**d**). The measured cross-polarization spectra show the resonant scattering peak, TE_01_ mode, red-shifting with NW diameter. The low intensity band in all spectra is the HE_11_ mode. The sinusoidal curve riding the measured spectra is an artifact.

Figure 4 shows the measured cross-polarization and reflectance spectra, and their simulated counterparts for varying NW average diameter. In the measured cross-polarization spectra (a), a peak is seen red-shifting with increasing NW diameter. The peak is due to resonant scattering from the NWs, which we assign to the TE_01_ optical mode as will be discussed below. The low intensity plateau below it can in turn be assigned to the HE_11_ mode^9,10,28–30^. Note that the periodical modulation on top of the measured spectra are caused by interference in the experimental setup. The horizontal streaks are due to uncertainty in the measurement of NW diameter. The signal detected at long wavelengths in Fig. 4(a), beyond the valley due to the HE_11_ resonance, is assigned to stray scattering from the NWs, which could originate from the leaky TM_01_ mode^25,26,31^.

A similar valley due to the HE_11_ mode is the most prominent feature in the measured reflectance (Fig. 4(b)), though the valley is noticeably wider compared to that in the cross-polarization measurements. We find also an indication of the TE_01_ mode in the reflectance but with weaker visibility than in cross-polarization. Thus, it can be concluded that cross-polarization method is a more precise method than reflectance to determine the likely position of the HE_11_ resonance from the narrower valley, and above all, cross-polarization allows us to directly see resonant scattering from the TE_01_ mode.

The SMM simulated cross-polarization spectra in Fig. 4(c) match well to the measured spectra and show the same features. Similarly, the simulated reflectance in Fig. 4(d) matches nicely to the measured reflectance. Thus, electromagnetic optics modeling appears suitable for investigating the origin of the cross-polarization. It was also noted that an increase in the array period causes the peak to red-shift slightly (see Supporting Information Figure S3(a)). The NW length, on the other hand, had only a negligible effect to the spectra (see Supporting Information Figure S3(b)).

**Figure 5:**
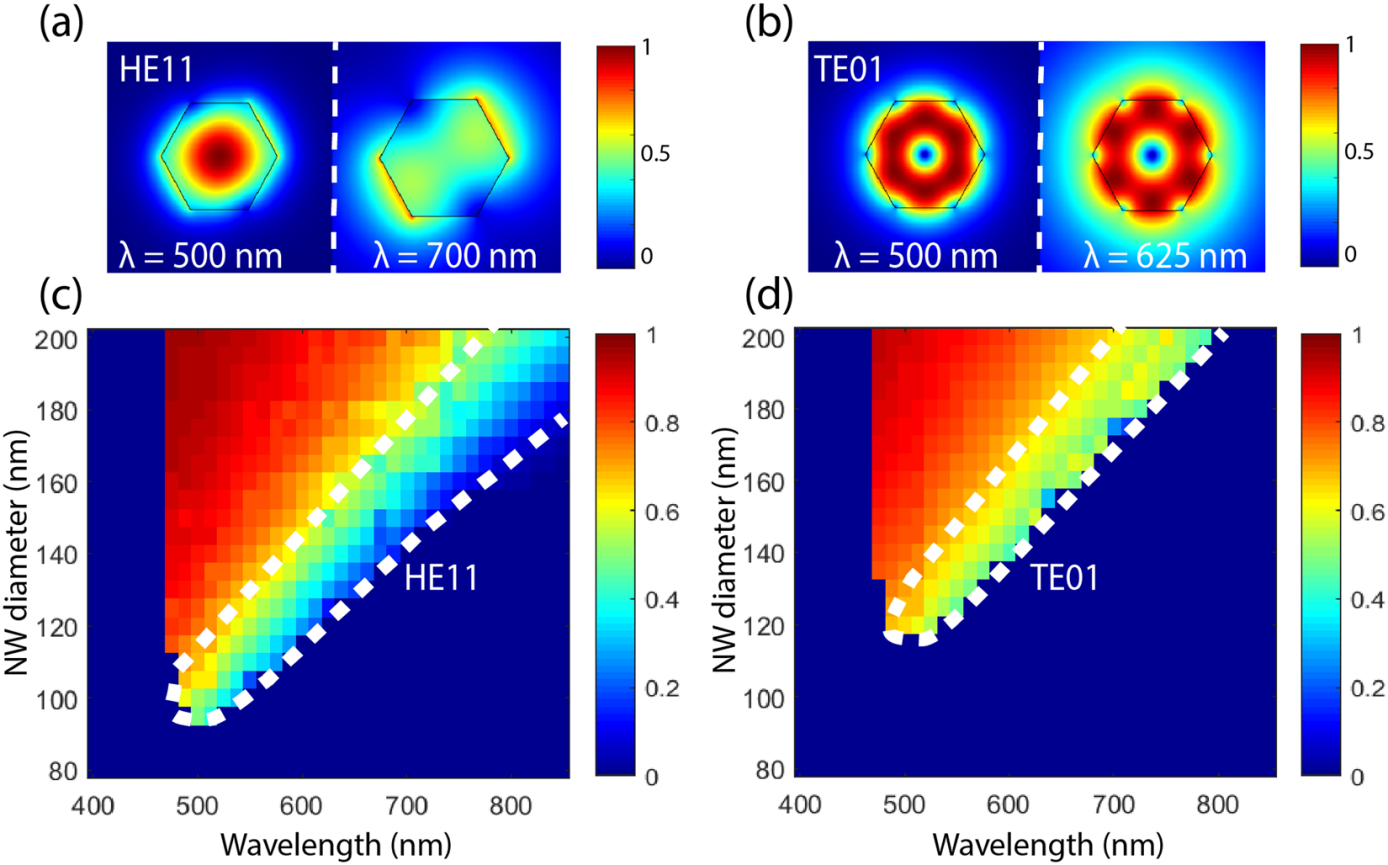
FEM simulated electric field distribution of HE_11_ (**a**) and TE_01_ (**b**) modes (NW diameter = 150 nm) illustrating how the mode is well confined within the NW at shorter wavelengths whereas at longer wavelengths the mode overlaps with the surrounding medium. The simulated color intensity maps showing where the HE_11_ (**c**) and TE_01_ (**d**) exist as guided modes. The color indicates the ratio of electric field distribution inside and outside the NW. The dashed areas denote areas where the modes have electric field partly outside the NW. These areas match to the measured and simulated bands in the cross-polarization spectra.

For further analysis, the guided optical modes in the InGaAs NWs were found with FEM modeling. Figure 5 shows color maps of the guiding ratio of the HE_11_ (a) and TE_01_ (b) modes with varying NW diameter and wavelength. The guiding ratio is defined here as the ratio of the electric field distribution inside the NW to the total electric field distribution, that is, both inside and outside the NW. Red color in the map corresponds to the electric field being mostly inside the NW and dark blue to it being mostly outside, as in the case of the HE_11_ mode, or to the mode not showing up at all, as is the case for the TE_01_ mode at long wavelengths.

For both modes, the wavelength region where the field is noticeably both inside and outside the NW (dashed region in the figure) red-shifts with the NW diameter in the same way as in the measured and simulated spectra (Fig. 4(a) and (c)). Furthermore, the dashed areas match nicely to the high intensity band, that is, the peak, for the TE_01_ mode and to the red-shifted low intensity band, that is, the dip, for the HE_11_ mode in the measured cross-polarization spectra (Fig. 4(a)). We explain the reason for this matching of localization/delocalization of a mode and resonant spectral features as follows: The coupling of incident light into a mode is more efficient when there is a large overlap between the incident light and the mode, which occurs when the mode is delocalized from the NW, i.e., it lies mostly outside of the NW. At the same time, the NW affects the mode more when the mode is localized within the NW. Then, optimal condition for light scattering from the NW is in between localized and delocalized to the nanowire, that is, when the electric field distribution is at the same time noticeably both inside and outside the NW^27^. In our modal analysis, only the HE_11_ mode and the TE_01_ mode show such behavior in the wavelength region that covers the reflectance dip and the cross-polarization peak. (See the Supporting information of ref.^32^ for analysis of a similar effect of mode overlap and localization to the nanowire in absorption resonances).

To explain why the TE_01_ mode is observed to radiate with high intensity and the HE_11_ mode with low intensity in the cross-polarization experiments, we turn to study the symmetry of the optical modes and the incident light. First, light at normal incidence, does not overlap with the TE_01_ mode. The electric field of the incident light is constant both in amplitude and phase in the cross-sectional plane of the NW array, whereas the TE_01_ has an electric field that rotates around the NW axis. This leads to zero overlap in terms of integration over the x-y plane. With increasing polar angle, the phase difference of the incident light over the cross-section of the NW can allow for coupling. Indeed, the cross-polarization, which we assign to coupling to the TE_01_ mode, is zero at normal incidence and increases with increasing polar angle (see Supporting Information Figure S4). However, the coupling to the TE_01_ mode is also polarization dependent. Only the TE component of the incident light is expected to couple efficiently to the TE_01_ mode, whereas the TM component is not expected to couple efficiently to it. The x-y component of the electric field of TM polarized incident light is parallel to the x-y component of the k-vector of the incident light (see Fig. 1(b) for a schematic of polarization definitions). This leads to zero overlap with the rotating field of the TE_01_ mode. At the same time, the linearly polarized light through the NA in the experiments leads to a linear combination of TE and TM polarized incident light, where the fraction of TE and TM polarization depends on the azimuth angle *ϕ* (in the modeling of the x-polarized incident light, the light is purely TM polarized for *ϕ* = 0 and purely TE polarized for *ϕ* = 90°).

Hence, the resulting cross-polarization originates from the underlying difference between how the TE and TM polarized components of the incident light can couple into the TE_01_ mode. And, on the fact that the linear polarizer in combination with the NA gives rise to both TE and TM polarized components to the incident light. Indeed, from modeling, we find the strongest cross-polarization for an azimuth angle of 45°, which shows the strongest mixing between TE and TM polarization (Supporting Information Figure S4). In contrast, both the TE and TM polarized components of the incident light can couple to the HE_11_ mode, and apparently we don’t find cross-polarization from the mode.

The source of cross-polarization from the NWs could in principle also be caused by other phenomena than scattering. Optical activity and photoluminescence (PL) were ruled out, see Supporting Information Figures S5 and S6.

One interesting avenue for further study of cross-polarization spectroscopy would be to study less absorbing NWs, like GaP NWs (according to SMM simulations, the cross-polarization signal is expected to be much stronger in a non-absorbing NW; see Supporting Information Figure S7). Such a study would be especially interesting if the incident polar and azimuth angle was controlled in a goniometer setup using collimated light.

## Conclusions

An optical microscopy method was presented to characterize experimentally optical modes from vertical NWs. The direct reflected light was filtered out with a crossed polarizer to detect the polarization converted scattered light only from the NWs. A peak in the cross-polarized scattered light was assigned to the TE_01_ mode, and the mode was observed to red-shift with increasing NW diameter. Also the HE_11_ mode was observed, but as a low intensity band that red-shifts in a similar way. Cross-polarization spectroscopy is a more precise method to determine the spectral location of the HE_11_ mode compared to conventional reflectance. Also, it allows the direct detection of the TE_01_ mode with good intensity, which is otherwise difficult with regular reflectance spectroscopy.

The modes were identified from FEM simulations. The observed resonances of the modes coincided with wavelength vs. diameter combinations where the mode had its electric field distribution noticeably both inside and outside the NW. In such a case, incident light can couple efficiently into the mode while at the same time interact strongly with the NW. The reason why the TE_01_ mode showed a peak in the cross-polarization experiments, while the HE_11_ mode didn’t, was assigned to the different symmetries of the modes relative to the incident light.

The presented method is beneficial when experimental determination of both the HE_11_ and the TE_01_ mode is needed. Such determination is directly helpful in the characterization of NW lasers where mode control is crucial for the application. Also, experimental assessment of the underlying optical modes of the NWs is helpful in the characterization of NW solar cells and LEDs, which usually have complex structures making highly predictive simulations cumbersome.

## Electronic supplementary material

Supporting Information
