# Supplementary material for: Measurement of Nanowire Optical Modes Using Cross-Polarization Microscopy
Source: Sci Rep. 2017 Dec 19;7:17790. doi: 10.1038/s41598-017-18193-1 (PMC5736640; doi:10.1038/s41598-017-18193-1)
Supplement: Supplementary file 1 — Supporting Information [file 41598_2017_18193_MOESM1_ESM.pdf]

# Supporting information for

## Measurement of Nanowire Optical Modes Using Cross-Polarization Microscopy

Joona-Pekko Kakko, Antti Matikainen, Nicklas Anttu, Sami Kujala, Henrik Mäntynen,Vladislav Khayrudinov, Anton Autere, Zhipei Sun, and Harri Lipsanen

Department of Electronics and Nanoengineering, Aalto University P.O.Box 13500, FI-00076 Aalto, Finland

### Sample diameters as a function of array period

Figure S1 plots the NW average diameters for each patterned hole diameter as a function of array period. The error bars on each data point mark the statistical deviation. An increasing trend of the NW diameter is visible with the array period. In the scattering matrix method (SMM) modeling, the modeled diameter was taken as $\sqrt{3\frac{\sqrt{3}}{2\pi}}$ times this hexagonal diameter to give the same area for the circular cross-section in SMM as for the corresponding hexagonal cross-section.

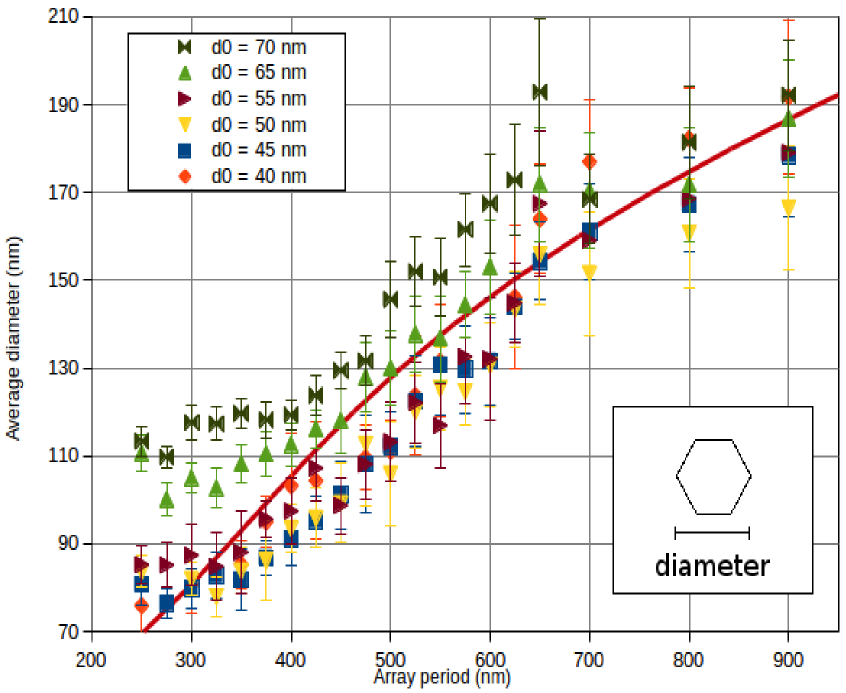


*Figure S1: Average diameters of NW arrays for each patterned hole diameter as a function of array period. The inset shows how the diameter was defined.The red solid line shows the fitting used in the SMM modeling to relate diameter to period.*

## Measured cross-polarization spectra with varied numerical aperture (microscope objective)

Figure S2 shows measured cross-polarization spectra with varying numerical aperture, or microscope objective, from NWs with average diameter (D) of 124.6 nm and array period (a) of 575 nm. The positions of the peaks do not shift when the numerical aperture changes, thus the colors perceived are unchanged with different magnifications.

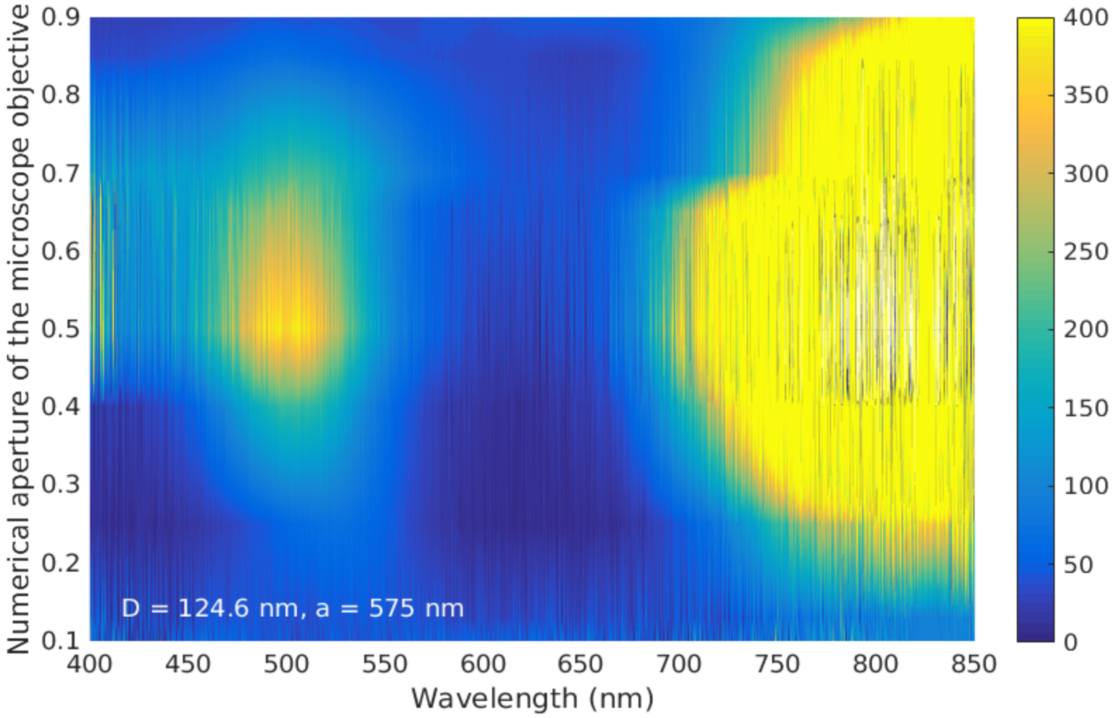


*Figure S2: Cross-polarization spectra with varying numerical aperture. The peak positions are unchanged when numerical aperture varies.*

## SMM simulated cross-polarization with varied array period and NW length

Figure S3 shows scattering matrix method (SMM) simulated cross-polarization spectra with varied array period and NW lengths. The increasing period causes the spectra to red-shift. In the studied range of NW lengths in the work (2 µm – 3 µm), no noticeable shift is seen.

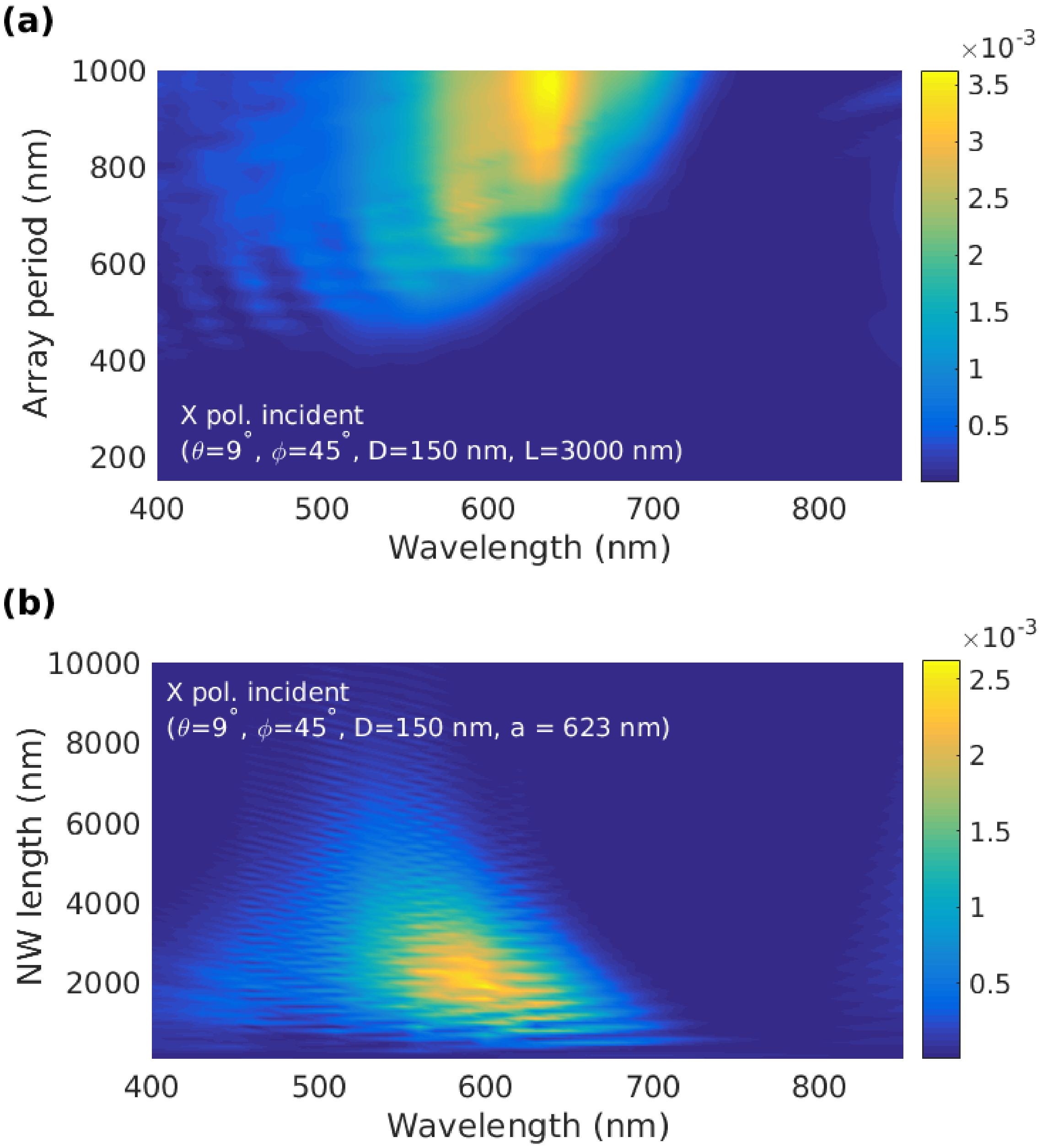


*Figure S3: Simulated cross-polarization spectra with varied array period (a) and NW length (b). A red-shift is visible with increasing array period.*

## SMM simulated cross-polarization with varied polar and azimuth angle

Figure S4 shows SMM simulations of the cross-polarization spectra with varying azimuthal $\varphi$ (a) and polar $\theta$ (b) angles. It is visible that when the azimuthal angle is 0˚ (90˚) the cross-polarization signal is zero, due to incident light being purely TM (TE) polarized, that is when there is no magnetic (electric) field component in the incidence plane. When the polar angle is zero, no cross-polarization is detected as the light cannot couple into the $TE_{01}$ mode. The cross-polarization signal is at its highest at the maximum collection angle (~14˚). The simulation with varying polar angle is in good agreement with measured cross-polarization spectra with varying numerical aperture in figure S2. With low polar angles, i.e., low numerical aperture, the cross-polarization signal vanishes.

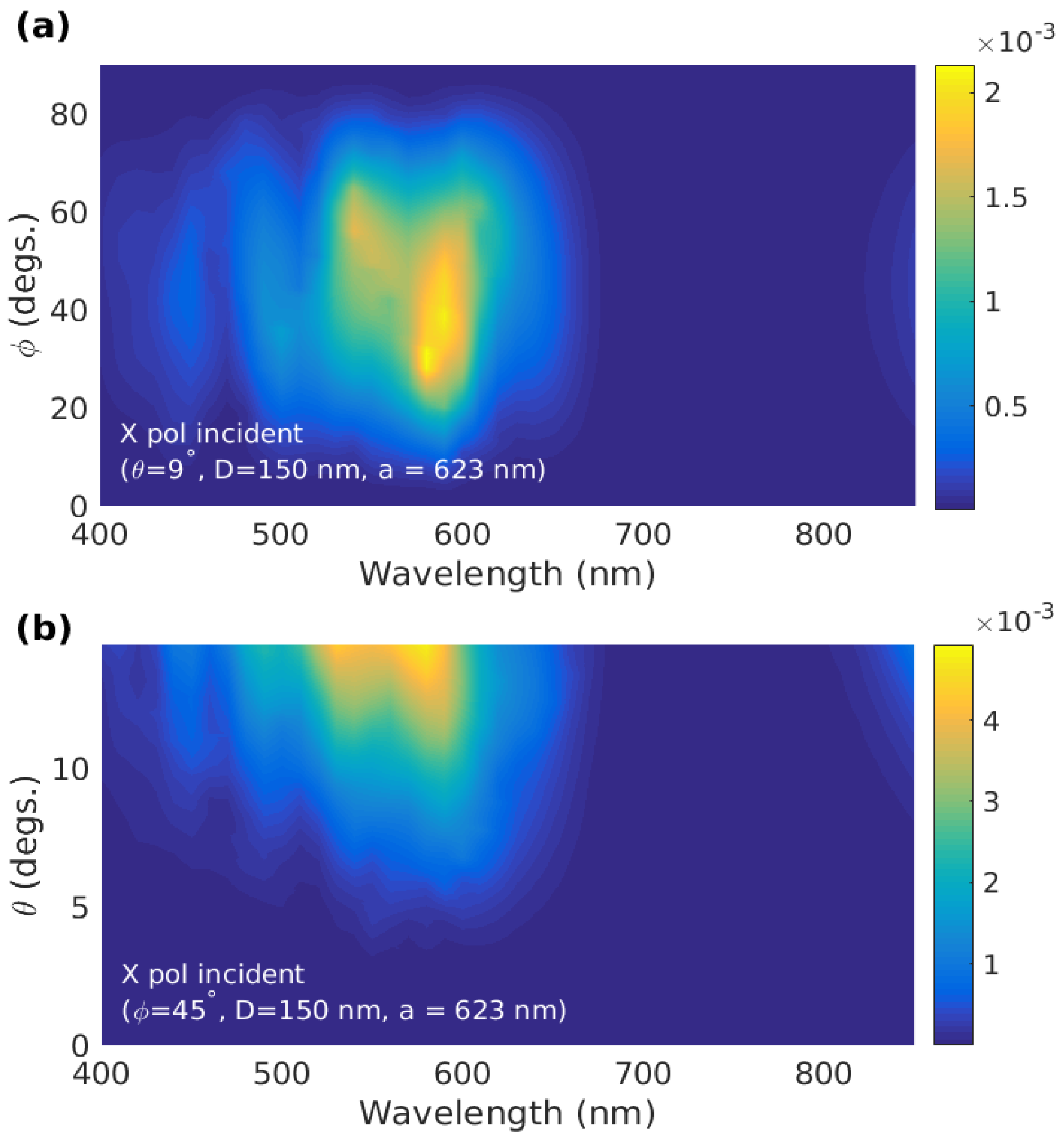


*Figure S4: Simulated cross-polarization spectra with varied azimuthal (a) and polar angle (b). No cross-polarization at azimuthal angles of 0˚ or 90 or when light is incident parallel to NW axis.'D' stands for diameter and 'a' for array period.*

## Cross-polarization spectra with sample rotation

Optical activity was studied from the InGaAs NWs by measuring the cross-polarization spectrum while the sample was rotated. Figure S5 shows a map of the spectra. No change in the peak position or in the intensity was seen with the rotation from 0° to 90°. Hence, the NW arrays are not optically active. This was also verified by rotating the analyzer to find a signal minimun, however, the signal minimum was still in the original analyzer position.

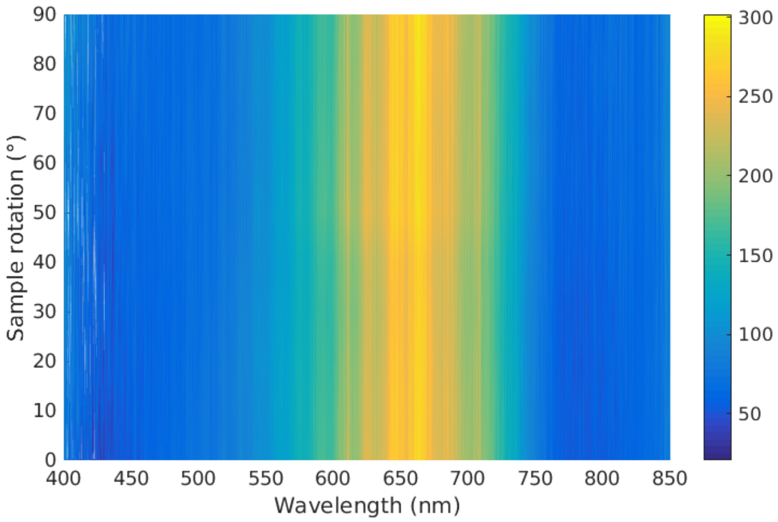


*Figure S5: Map of the cross-polarization spectra with varying sample rotation. No change in the peak position or intensity is visible. The average NW diameter was 169 nm and array period 800 nm. The color corresponds to signal relative to substrate in percents.*

## Low-temperature photoluminescence from InGaAs NWs

The photoluminescence (PL) of the InGaAs NWs was measured with an excitation wavelength of 532 nm. The spot size was approximately 400 µm and the optical power incident to the sample was approximately 3 mW. The signal was acquired using a monochromator and a liquid nitrogen cooled Ge detector (Teledyne Judson). Lock-in amplifier was used to amplify the signal. Figure S6 shows PL spectra measured at temperature range from 20 K to 50 K. The PL signal quenched quickly as the temperature was increased.

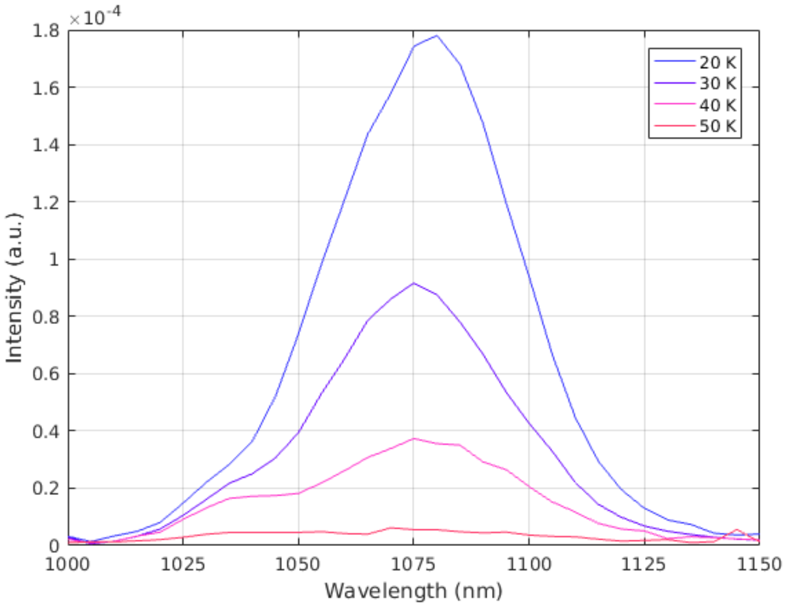


*Figure S6: Low-temperature PL spectra of InGaAs NWs with varying temperature*

## SMM simulated cross-polarization for non-absorbing NWs

Figure S7 shows SMM simulated cross-polarization spectra for non-absorbing NWs ($Im(n_{InGaAs})$ = 0). An order of magnitude increase in signal is seen compared to absorbing NWs. The $TE_{01}$ band is much more visible in these non-absorbing NWs. The change in the dispersion of the band at $\lambda<500$ nm is due to anomalous dispersion of $Re(n_{InGaAs})$ there[1].

Note that the $HE_{11}$ band is not distinguishable here: For the absorbing NWs in the main text, the $HE_{11}$ mode caused a dip between the peak due to the $TE_{01}$ mode and the stray cross-polarization at long wavelengths. In these non-absorbing NWs, the peak due to the $TE_{01}$ mode was enhanced, but the long-wavelength stray background was not enhanced, making the identification of a possible dip due to the $HE_{11}$ mode non-trivial.

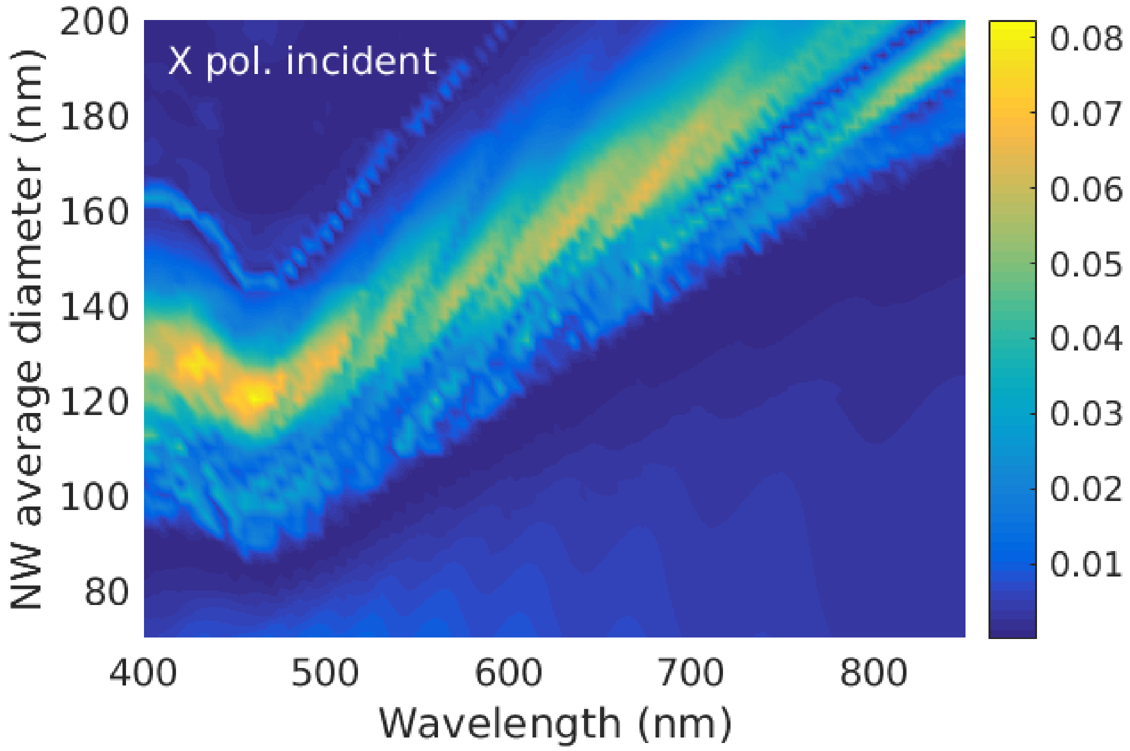


*Figure S7: SMM simulated cross-polarization spectra for non-absorbing NWs shows an order of magnitude stronger signal compared to the measured NWs.*

1 N. Anttu, S. Lehmann, K. Storm, K. A. Dick, L. Samuelson, P. M. Wu, and M.-E. Pistol, Nano Letters 14, 5650 (2014).
